# Is It Possible for Patients with Early Distal Junctional Kyphosis following Adult Cervical Deformity Corrective Surgery to Achieve Similar Outcomes to Their Unaffected Counterparts? An Analysis of Recovery Kinetics

**DOI:** 10.3390/jcm13113246

**Published:** 2024-05-31

**Authors:** Oluwatobi O. Onafowokan, Bailey Imbo, Tyler Williamson, Ankita Das, Jamshaid M. Mir, Matthew Galetta, Nathan Lorentz, Peter G. Passias

**Affiliations:** 1 Division of Spinal Surgery, Departments of Orthopaedic and Neurosurgery, NYU Langone Medical Center, NY Spine Institute, New York, NY 10003, USA; 2 Department of Orthopaedic Surgery, University of Texas Health San Antonio, San Antonio, TX 78229, USA

**Keywords:** cervical deformity, alignment, distal junctional kyphosis, recovery kinetics

## Abstract

**Background:** Distal junctional kyphosis (DJK) is a concerning complication for surgeons performing cervical deformity (CD) surgery. Patients sustaining such complications may demonstrate worse recovery profiles compared to their unaffected peers. **Methods:** DJK was defined as a >10° change in kyphosis between LIV and LIV-2, and a >10° index angle. CD patients were grouped according to the development of DJK by 3M vs. no DJK development. Means comparison tests and regression analyses used to analyze differences between groups and arelevant associations. **Results:** A total of 113 patients were included (17 DJK, 96 non-DJK). DJK patients were more sagittally malaligned preop, and underwent more osteotomies and combined approaches. Postop, DJK patients experienced more dysphagia (17.7% vs. 4.2%; *p* = 0.034). DJK patients remained more malaligned in cSVA through the 2-year follow-up. DJK patients exhibited worse patient-reported outcomes from 3M to 1Y, but these differences subsided when following patients through to 2Y; they also exhibited worse NDI (65.3 vs. 35.3) and EQ5D (0.68 vs. 0.79) scores at 1Y (both *p* < 0.05), but these differences had subsided by 2Y. **Conclusions:** Despite patients exhibiting similar preoperative health-related quality of life metrics, patients who developed early DJK exhibited worse postoperative neck disability following the development of their DJK. These differences subsided by the 2-year follow-up, highlighting the prolonged but eventually successful course of many DJK patients after CD surgery.

## 1. Introduction

Adult cervical deformity (CD) is a complex pathology characterized by the interruption of the normal cervical vertebral alignment in the sagittal and/or coronal planes [1,2]. CD is of heterogenous etiology, with the potential to cause severe discomfort and disability, and is also associated with poor health-related quality of life metrics [3]. Surgical intervention for CD can provide affected patients with significant improvements in quality of life [4,5]. However, it is a complex surgery and is associated with considerable complication and revision rates [2,5].

Distal junctional kyphosis (DJK) is a mechanical failure complication which remains of particular concern following surgical correction for CD, and is a frequent reason for revision surgery [6,7]. DJK denotes a progression in the degree of kyphosis of the vertebral segment adjacent to the lower instrumented vertebra postoperatively [8]. DJK can result in considerable morbidity, including pain, imbalance, and degenerative disc pathology due to increased mechanical stress on adjacent vertebral segments [9,10]. The development of early DJK (within three months postoperatively) is associated with particularly more severe radiographic malalignment and neurologic decline [11]. To our knowledge, there is limited information on the effect of early postoperative DJK on CD surgical recovery.

Mechanical failure complications following surgery to the thoracolumbar spine, such as proximal junctional kyphosis and proximal junctional failure, have been well studied and a body of literature exists which provides strategy for preventing such complications and identifying particularly at-risk patients [12,13]. DJK, which is the more likely mechanical complication following CD surgery, has not been studied as extensively. In this context, this study aims to investigate the recovery course following CD surgery in patients who develop early DJK, particularly examining the variation in health-related quality of life metrics up to two years postoperatively.

## 2. Methods

### 2.1. Study Design

This was a retrospective cohort study of adult cervical deformity (CD) patients aged 18 years and older who were prospectively enrolled into a single-center registry between 2012 and 2019. Institutional Review Board (IRB) approval was obtained prior to enrolment and all included individuals provided informed consent. CD was defined as ≥1 of the following: C2–C7 sagittal kyphosis > 15°; T1 slope–cervical lordosis mismatch (TS-CL) > 35°; C2–C7 sagittal vertical axis (cSVA) > 40 mm; chin-brow vertical angle (CBVA) > 25°; McGregor’s slope (MGS) > 20°; or segmental cervical kyphosis > 15° across any 3 vertebrae between C2 and T1. Patients included in the present study underwent surgical intervention for CD and had complete demographic, radiographic, and health-related quality of life (HRQL) data at baseline and up to at least 2 years preoperatively. Patients who underwent revision surgery for any reason were excluded.

Indications for surgery included the following: neurological deficit, persistent severe pain despite conservative measures, spondylotic myelopathy, functionally limiting postural deformity, and airway and/or esophageal compromise. Distal junctional kyphosis was defined by the development of an angle <−10° from the distal end of the fusion construct to the second adjacent distal vertebra, and/or a change in this angle by <−10° from baseline [14]. “Early DJK” denoted patients developing this complication by three months postoperatively. No patients included in this study underwent revision for DJK or any other reason.

### 2.2. Data Collection

Demographic, radiographic, surgical, and HRQL data were collected. HRQL data collected preoperatively and at all follow-up timepoints include the Neck Disability Index (NDI), Numeric Rating Scale for the neck (NRS-Neck), EuroQol-5 Dimension (EQ-5D), and modified Japanese Orthopaedic Association (mJOA) assessment. The minimally clinically important difference (MCID) for the mJOA was set at 2 based on published values [15,16]. The MCID for NDI was set as 15, which is double the published MCID value, due to our employed NDI score being collected on a 0–100 scale as opposed to 0–50 [17,18]. The NRS-Neck MCID was set as 2 as per previously published values [17,19].

Lateral erect spine radiographs were used to assess radiographic parameters at baseline and follow-up intervals. All images were analyzed with SpineView^®^ (ENSAM, Laboratory of Biomechanics, Paris, France). Spinopelvic radiographic parameters assessed included pelvic tilt (PT), pelvic incidence–lumbar lordosis mismatch (PI-LL), and the sagittal vertical axis (SVA). Cervical spine parameters assessed included cervical lordosis (C2–C7 angle), cervical sagittal vertical axis (cSVA: C2 plumb line relative to the posterosuperior corner of C7), T1 slope (T1S), C2 slope (C2S), T1 slope minus cervical lordosis (TS-CL), and McGregor’s slope (MGS).

### 2.3. Development of the Normalized Integrated Health State

**Figure 1:**
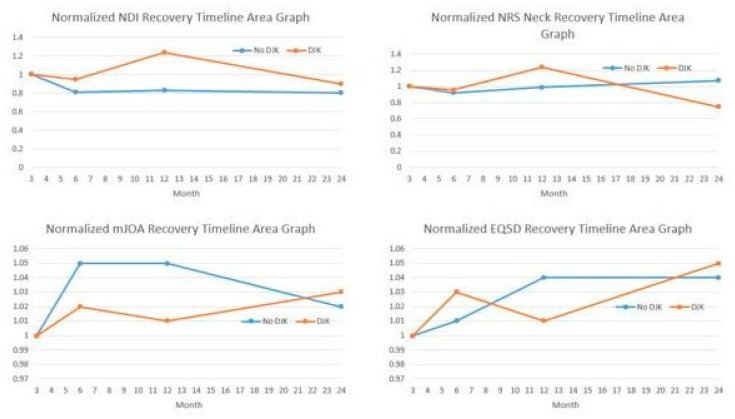
Illustration of recovery kinetics in different patient-reported outcome metrics. EQ5D = EuroQol 5-domain questionnaire; mJOA = modified Japanese Orthopaedic Association; NDI = Neck Disability Index; NRS = Numeric Rating Scale.

Normalized HRQLs were developed and analyzed, permitting the calculation of an integrated health state using the following validated novel area-under-the-curve methodology [20,21]. Collected HRQL metrics at any postoperative timepoint (e.g., 3-month, 6-month, 1-year, and 2-year) were divided by the corresponding preoperative score for each patient. The resulting preoperative normalized HRQL score for all patients was therefore 1, with any follow-up normalized HRQL score being >1, equal to 1, or <1, corresponding to whether the patient improved or deteriorated relative to baseline. Normalized HRQL scores were then plotted on an area graph, with the *x*-axis representing time (in months, starting at the preoperative interval) and the *y*-axis representing normalized HRQL scores (Figure 1). Regarding Integrated Health State (IHS) values for varying outcome metrics, lower NDI IH, lower NRS neck his, and higher mJOA IHS scores indicated a better outcome (better recovery process).

### 2.4. Statistical Analysis

Means comparisons tests (*t*-tests and ANOVA) were used to analyze collected variables, with Pearson chi-square tests used for categorical variables. Multivariable analyses (ANCOVA) were used to determine the differences between groups in achieving the MCID in HRQL score improvements while factoring any baseline and perioperative differences. Multivariable logistic regression analysis assessed associations between DJK development and changes in HRQL outcomes. All analyses were performed using SPSS software (v28.0, IBM Armonk, NY, USA), with significance set to *p* < 0.05. 

## 3. Results

### 3.1. Cohort Overview

There were 113 patients included in this study. The mean age was 61.1 ± 16.3 years, the mean body mass index (BMI) was 27.1 ± 5.7 kg/m^2^, and the mean Charlson Comorbidity Index (CCI) was 0.75 ± 0.5. In total, 65% of patients were female.

### 3.2. Surgical Descriptors

The mean amount of levels fused was 5.2 ± 3.5, the mean estimated blood loss (EBL) was 894 ± 564 mL, and the mean length of operation was 405.0 ± 185.1 min. By surgical approach, 7.0% of patients underwent an anterior-only approach, 59.7% underwent posterior-only approach, and 31.3% underwent a combined approach. The most common upper instrumented vertebra (UIV) was C3, and the most common lower instrumented vertebra (LIV) was C7. Overall, 60.4% underwent an osteotomy as part of their procedure (Table 1). DJK patients demonstrated more severe malalignment in cSVA and CBVA at baseline (Table 2). There were no differences in other measured radiographic parameters.

### 3.3. Postoperative Distal Junctional Kyphosis

Of the 113 patients included in the analysis, 17 developed DJK and 96 did not. Comparing those that developed DJK and those who did not, age (60.3 vs. 62.2), gender (F: 71.0% vs. 61.0%), BMI (27.0 vs. 28.3 kg/m^2^), CCI (0.77 vs. 0.98), operating time (484.0 vs. 556.5 min), EBL (1028.3 vs. 843.9 mL), and the presentation of neurologic symptoms (70.6% vs. 76.0%) were similar between groups (*p* > 0.05). Patients who developed early DJK had more severe preoperative deformity (cervical sagittal vertical axis {cSVA}: 59.0 vs. 43.9 mm, *p* = 0.031), underwent more osteotomies (76.5% vs. 49.0%, *p* = 0.005), and underwent more combined approaches (64.7% vs. 26.0%, *p* = 0.002). There were no statistically significant differences between the two groups with regard to posterior approaches, decompressions, and the amount of levels fused. Following surgery, the rate of complications and the development of neurological symptoms were similar between groups, except that DJK patients experienced more dysphagia (17.7% vs. 4.2%; *p* = 0.034). 

### 3.4. Recovery Kinetics 

There were no significant differences between DJK and non-DJK patients at baseline in NDI, NRS-Neck, mJOA, and EQ5D scores. Non-DJK patients generally trended towards better HRQL scores at 1 year, with no significant differences at 2 years (Table 3). Radiographic metrics similarly did not differ between the groups at the 2-year follow-up (Table 4). DJK patients exhibited worse neck disability (NDI) Integrated Health State recovery from 3 months to 1 year, but these differences subsided when following patients through 2 years (Figure 1). DJK patients had worse NDI, NRS, and mJOA scores at 1 year, but these differences had subsided by the 2-year follow up (Figure 1). Non-DJK patients had higher rates of achieving the MCID in NDI at 3 months (39.5 vs. 28%, *p* = 0.031) and at 1 year (44 vs. 35%, *p* = 0.043). However, there were no significant differences at 2 years (46.2 vs. 39.7%, *p* = 0.051). Similar trends in the MCID for NRS neck scores were seen at 3 months (67.3 vs. 46%, *p* = 0.012) and 1 year (65.2 vs. 49%, *p* = 0.033), but not at 2 years (64% vs. 55%, *p* = 0.054). There were no significant differences between DJK and non-DJK patients with regard to the MCID in the mJOA score at all timepoints. Logistic regression analyses controlling for preop deformity (by cSVA magnitude) and surgical invasiveness (osteotomy and combined approach use) revealed that patients experiencing DJK were more likely to experience worsening from baseline NDI score postoperatively by 3 months (OR 2.11, 95% CI: 1.36–5.81) and at 1 year (OR 1.25, 95% CI: 1.05–1.49, *p* = 0.035). These trends were not seen for NRS, mJOA, and EQ5D scores.

## 4. Discussion

The frequency of surgical intervention for CD surgery is increasing due to advancements in technique and patient selection [22]. With the increased frequency of cervical vertebra instrumentation, mechanical failure complications such as distal junctional kyphosis (DJK) are becoming more notable [10]. In the cervical spine specifically, DJK has been defined by the development of an angle <−10° from the distal end of the fusion construct to the second adjacent distal vertebra, and/or a change in this angle by <−10° from baseline [14]. DJK is an important issue to address as it can significantly impact the affected patients’ surgical journey, potentially resulting in increased overall cost and also deterioration in achieved clinical and radiographic improvements. Therefore, this study aimed to investigate the differences between patients developing postoperative DJK and their unaffected counterparts, with a view to assessing if DJK patients eventually experienced similar levels of improvements as the non-DJK patients.

**Figure 2:**
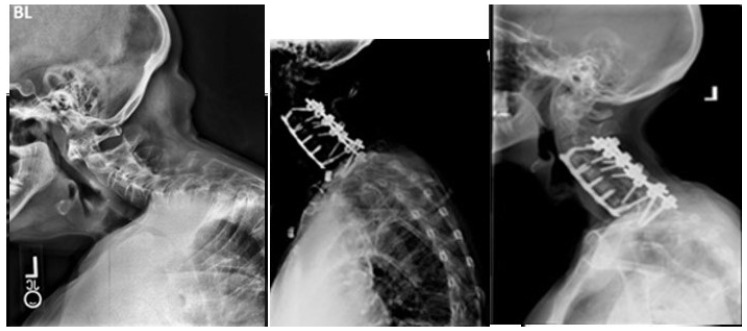
A 71-year-old female from the DJK group. Images from left-to-right: preoperative, immediate postoperative, and 3-month postoperative X-rays. History of progressive right-sided neck pain with progressive radiculopathy and myelopathy. Symptoms unresolved with conservative measures. Underwent C3–C7 ACDF with C3-T1 posterior fusion. Symptoms initially showed some improvement up to 6 weeks, before showing gradual worsening.

**Figure 3:**
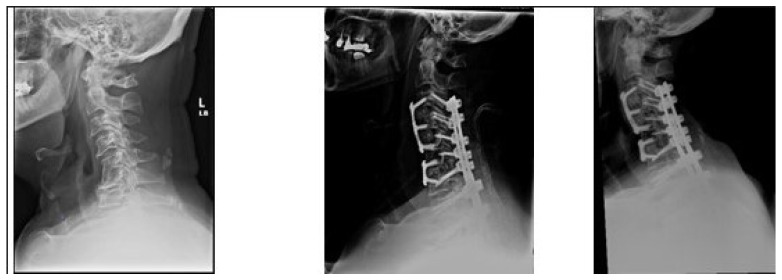
A 70-year-old male from the non-DJK group. Images from left-to-right: preoperative, immediate postoperative, and 3-month postoperative X-rays. History of intractable neck pain with sensory and motor right upper limb deficits. Had also previously undergone L2-to-pelvis fusion for degenerative lumbar disc disease. Underwent C3–C5 and C6–C7 ACDF with C3-T2 posterior fusion. Symptoms showed improvement and radiographic alignment was maintained without evidence of DJK.

Our study reports a DJK rate of 15% among the patients included in analysis. Perhaps unsurprisingly, patients who developed DJK had significantly worse cervical sagittal malalignment preoperatively (Figure 2 and Figure 3). Excess preoperative malalignment has previously been reported to be predictive of DJK development [9,10,14,23]. Passias et al. studied 101 patients undergoing CD surgery and reported that excessive preoperative malalignment beyond certain thresholds of cervical lordosis (<−12°, cSVA > 56.3 mm, and TS-CL > 36.4°) resulted in a five to six times increased risk for DJK [14]. Patients who developed DJK also underwent a significantly higher frequency of osteotomies and combined surgical approaches, compared to non-DJK patients. Combined surgical approaches and the Smith Peterson osteotomy have previously been reported as notable predictors of DJK [11,14].

Predictably, patients in our study who developed early DJK still exhibited significantly worse cervical sagittal malalignment (cSVA) than their unaffected counterparts at two years. Both preoperative and postoperative malalignment have been associated with increased rates of DJK [9,24]. These patients also exhibited consistently worse HRQL metrics (NDI and EQ-5D) at follow up until one year. Interestingly, these differences were insignificant at two years postoperatively. This indicates that despite these patients still displaying radiographic evidence of malalignment after two years, their overall levels of disability and symptomaticity had eventually improved to comparable levels with their non-DJK counterparts. This was especially evident in the patients who underwent revision surgery due to DJK, who also achieved comparable HRQL outcomes at two years. We have not been able to identify factors contributing to this improvement between one year and two years postoperatively. Previous studies into mechanical failure after cervical vertebra instrumentation predominantly involved follow-up until one-year postoperatively [6,9,10,14,24]. Future studies will need to include longer term follow-up in order to further shed light on this.

This study is not without limitations. The retrospective nature combined with relatively small sample sizes may limit the generalizability of findings. The relatively limited sample size may potentially result in restricted clinical variation and truncation in certain areas. Additionally, due to the heterogenous nature of CD, there is potential for limitations in the applicability of radiographic parameters employed in analyzing this pathology. The heterogeneous nature of CD does not allow for a more in-depth analysis of focal preoperative malalignments either. We have not included an analysis of additional therapeutic modalities used postoperatively either. Such an analysis may have shed some light on the HRQL improvements noted in the DJK patients between one and two years postoperatively.

## 5. Conclusions

Despite exhibiting similar preoperative health-related quality of life metrics, patients who developed early postoperative DJK exhibited worse postoperative neck disability following the development of their DJK, when compared with their unaffected counterparts. These differences had subsided by the 2-year follow-up, highlighting the prolonged but eventually successful course of many DJK patients after CD surgery without needing to undergo revision surgery.

## Figures and Tables

**Table 1:** Demographic and surgical factor comparisons.

	DJK	Non-DJK	Sig.
Age, years	60.3	62.2	0.355
Gender, % female	71% female	61% female	0.080
BMI, kg/m^2^	27.0	28.3	0.311
CCI	1.11	0.95	0.684
Levels fused	7.0	6.0	0.147
EBL, mL	1028.3	843.9	0.052
Operative length, mins	484.0	556.5	0.064
Osteotomies, %	76.5	49	0.005

BMI = body mass index, CCI = Charlson Comorbidity Index; EBL = estimated blood loss.

**Table 2:** Baseline radiographic comparisons.

Parameter	DJK	Non-DJK	*p*-Values
PT, °	12.8	19.0	0.188
PI, °	54	53.0	0.851
PI-LL, °	3.20	5.01	0.051
TK, °	−30.8	−16.8	0.071
SVA, mm	−18.9	−14.5	0.535
TS-CL, °	28	23	0.442
CL, °	−9.5	−4.5	0.117
cSVA, mm	59	43.9	0.031
CBVA, °	−9.5	−1	0.037
C2 slope, °	30.2	32.2	0.169

CBVA = chin-brow to vertical angle, CL = cervical lordosis; cSVA = cervical (C2–C7) sagittal vertical axis; PT = pelvic tilt; PI = pelvic incidence; PI-LL = pelvic incidence–lumbar lordosis mismatch; SVA = C7–S1 sagittal vertical axis; TK = T4–T12 thoracic kyphosis, TS-CL = T1 slope-cervical lordosis mismatch.

**Table 3:** Health-related quality of life metrics.

	DJK	Non-DJK	Sig.
NDI BL	54.9	57.6	0.099
NDI 1Y	45.6	38.2	0.046
NDI 2Y	40	37.2	0.289
NRS-Neck BL	7	6.5	0.743
NRS-Neck 1Y	4	4.4	0.048
NRS-Neck 2Y	6	4.7	0.162
mJOA BL	9.8	11.2	0.671
mJOA 1Y	11	14.5	0.023
mJOA 2Y	13.3	14.3	0.718
EQ5D BL	6.7	5.6	0.882
EQ5D 1Y	6.7	5.8	0.245
EQ5D 2Y	4.5	5.3	0.468

EQ5D = EuroQol 5 domain questionnaire; mJOA = modified Japanese Orthopaedic Association score; NDI = Neck Disability Index; NRS-Neck = Numeric Rating Scale score.

**Table 4:** Postoperative radiographic and complication comparisons.

Parameter	DJK	Non-DJK	*p*-Values
PT 1Y, °	19.2	20.4	0.844
PT 2Y, °	20.6	19.6	0.111
PI 1Y, °	52.9	58.6	0.501
PI 2Y, °	53.9	57.6	0.690
PI-LL 1Y, °	1.11	0.75	0.352
PI-LL 2Y, °	3.5	2.6	0.071
TK 1Y, °	−7.3	−8.1	0.665
TK 2Y, °	−7.5	−7.3	0.822
SVA 1Y, mm	3.36	3.58	0.993
SVA 2Y, mm	2.44	1.56	0.754
TS-CL 1Y, °	24	19.9	0.470
TS-CL 2Y, °	26.9	23.5	0.628
CL 1Y, °	4.7	6.2	0.332
CL 2Y, °	1.25	4.3	0.132
cSVA 1Y, mm	16.9	19.5	0.171
cSVA 2Y, mm	15.3	18.7	0.231
CBVA 1Y, °	−1.9	−1.3	0.075
CBVA 2Y, °	1.6	1.1	0.210
C2 slope 1Y, °	20.4	18.7	0.247
C2 slope 2Y, °	23.6	20	0.601
DJF 2Y, %	16.5	6.3	0.015
Neurologic complications 2Y, %	25.4	8	0.023

CL = cervical lordosis; cSVA = cervical (C2–C7) sagittal vertical axis; DJF = distal junctional failure; PT = pelvic tilt; PI = pelvic incidence; PI-LL = pelvic incidence–lumbar lordosis mismatch; SVA = C7–S1 sagittal vertical axis; TK = T4–T12 thoracic kyphosis; TS-CL = T1 slope-cervical lordosis mismatch.

## Data Availability

The data used in this study is not publicly available due to Health Insurance Portability and Accountability Act and Institutional restrictions.
